# Editor's note on ‘Depletion of TDP 43 overrides the need for exonic and intronic splicing enhancers in the human apoA-II gene’

**DOI:** 10.1093/nar/gkad854

**Published:** 2023-10-04

*Nucleic Acids Research*, Volume 33, Issue 18, 1 October 2005, Pages 6000–6010, https://doi.org/10.1093/nar/gki897

The Editors were alerted in July 2022 about potential issues with Figures 2C and 3B as detailed below.

- Figure 2C: ISE3wt SRp75 and ISE3wt SRp55 appear to be identical. ISE3m SRp75 and + SRp75 appear to be identical.
- Figure 3B: Lanes 5 and 6 appear to be identical.

An Expression of Concern was published in February 2023.

The experiments were conducted almost 20 years ago, and the authors no longer have the original data.

The Editors analysed the two figures and noted areas of similarity in Figure 2C, as well as a likely splice line between the last two lanes of Figure 3B. Some images resulting from the analyses are provided below.

**Figure 2C.**
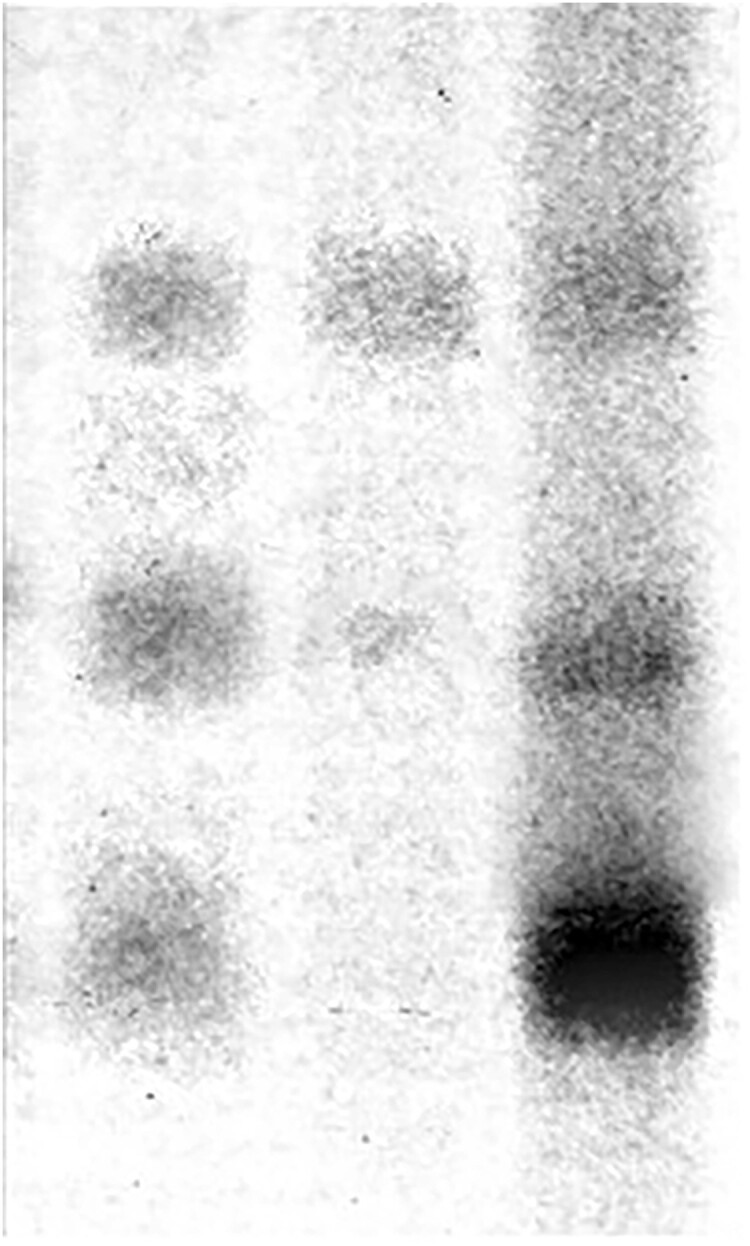
Unaltered published image.

**Figure 2C.**
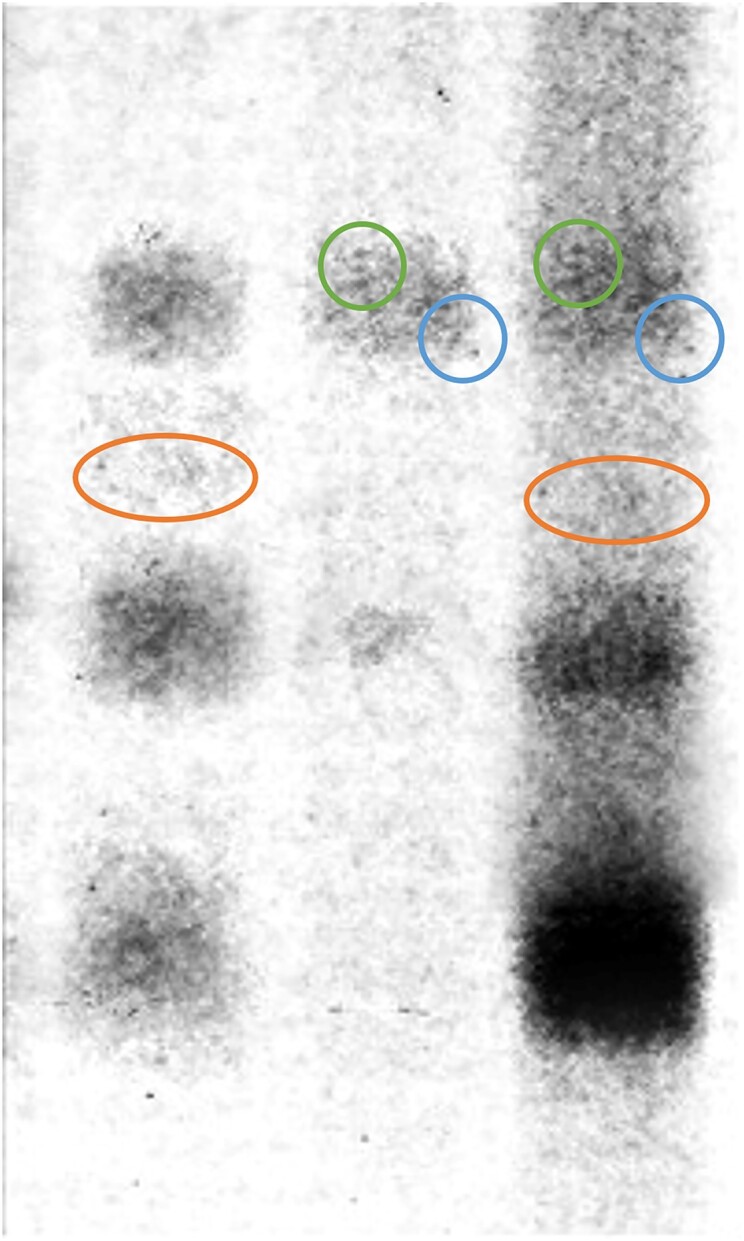
Published image after brightness and contrast adjustments. Circles of the same colour point to areas of similarity.

**Figure 2C.**
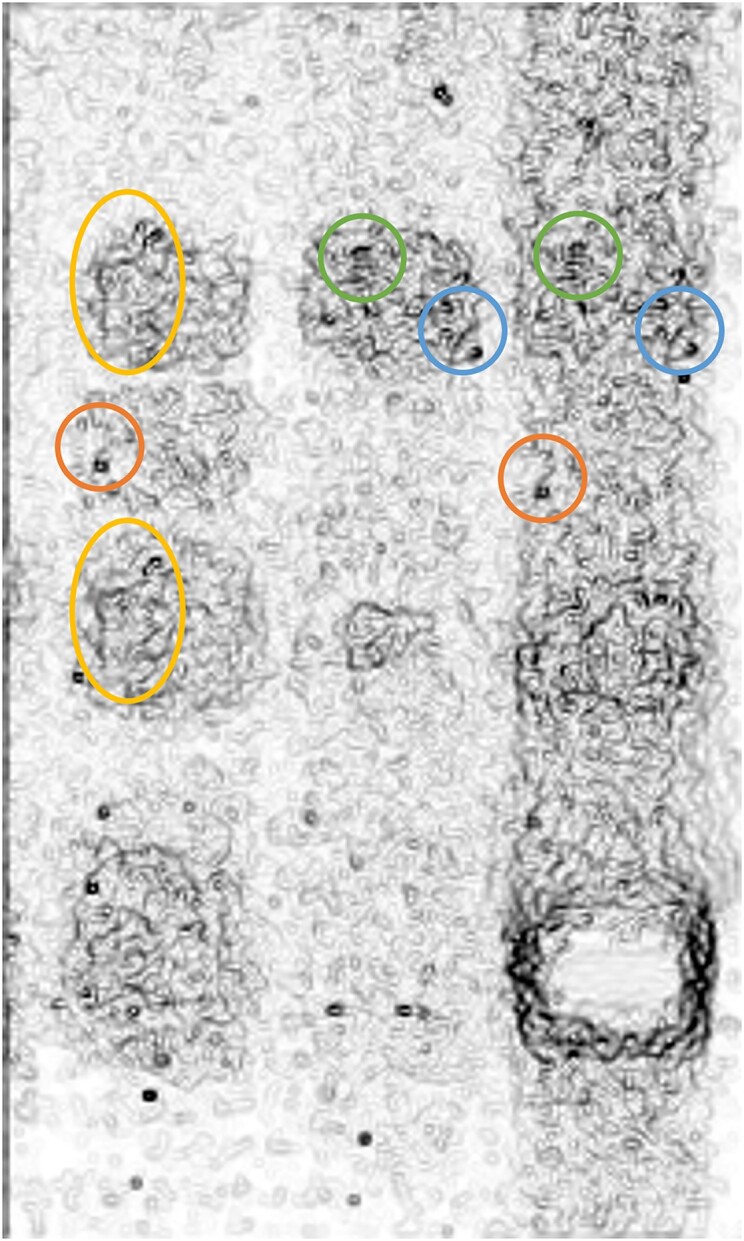
Published image after Edges filter. Circles of the same colour point to areas of similarity.

**Figure 3B.**
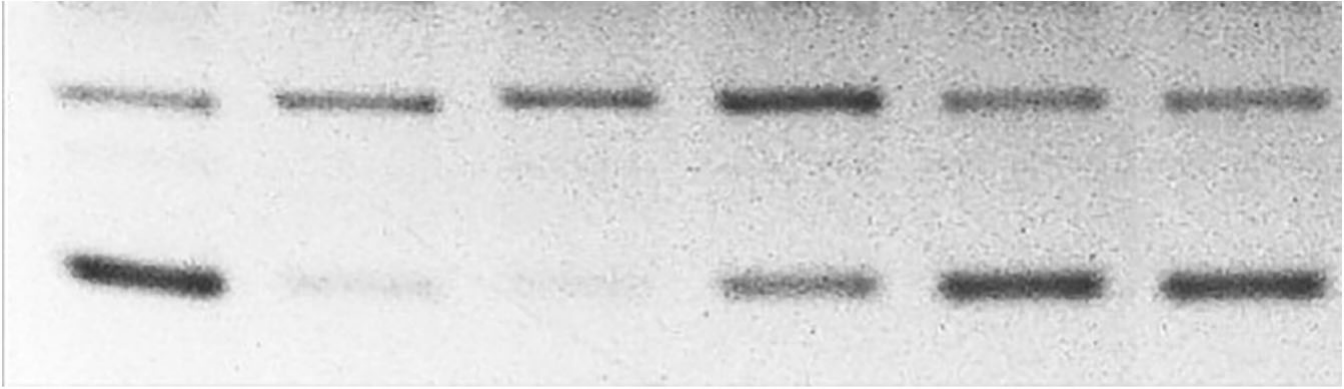
Unaltered published image.

**Figure 3B.**
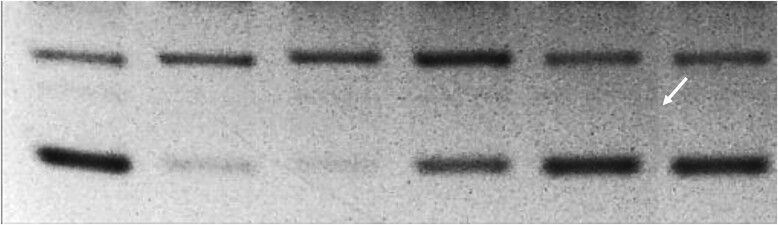
Published image after brightness and contrast adjustments. Arrow points to a possible splice line.

**Figure 3B.**
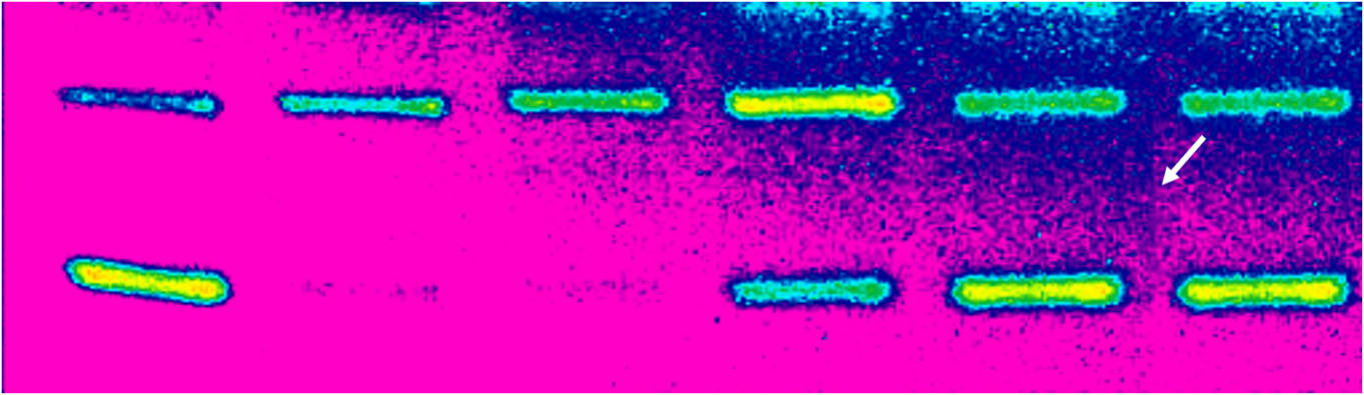
Published image after Gradient Map adjustment. Arrow points to a possible splice line.

The Editors referred the matter to the authors institution for investigation, however the institution referred the matter back to the authors. The authors maintain that they no longer have the original data. The Editors therefore do not have sufficient information to definitively refute the issues identified. While these issues may not affect the results or conclusion of the study, in the absence of original data, the Editors advise readers to examine Figures 2C and 3B with care.

**Julian E. Sale and Barry L. Stoddard**

Senior Executive Editors

